# Impact and Analysis of the Renovation Program of Dilapidated Houses in China on Poor Peasant Households’ Life Satisfaction: A Survey of 2617 Peasant Households in Gansu Province

**DOI:** 10.3390/ijerph192315548

**Published:** 2022-11-23

**Authors:** Tianyi Zhang, Qianqian Xu, Qi Zhang, Jun Wan

**Affiliations:** 1 School of Economics and Resource Management, Beijing Normal University, Beijing 100875, China; 2 China Institute for Poverty Reduction, Beijing Normal University, Beijing 100875, China; 3 China Rural Revitalization and Development Research Center, Beijing Normal University, Zhuhai 519087, China; 4 School of Banking and Finance, University of International Business and Economics, Beijing 100029, China

**Keywords:** Renovation Program of Dilapidated Houses, life satisfaction, living environment, PSM, Generalized Ordered Logit model

## Abstract

In developing countries, housing difficulties and environmental problems for poor peasants are prominent. In 2008, China began to explore pilot projects for the renovation of dilapidated houses in rural areas and has achieved remarkable results. This paper examines the impact of China’s Renovation Program of Dilapidated Houses on the life satisfaction of poor peasant households. Using firsthand survey data of four poverty-stricken counties in Gansu Province and the Propensity Score Matching method, we find that the program significantly improves the poor peasant households’ life satisfaction, with a greater impact on non-poverty-stricken villages and general-assurance households. In the mechanism analysis, we find that these positive treatment effects are mainly driven by the increases in wage income and operating income. In addition, we relax the assumption of parallelism and use the Generalized Ordered Logit model to further explore how this impact varies between groups. We show that people with a high level of life satisfaction are more significantly affected by the program. This study provides evidence that a sustainable living environment can improve the overall wellbeing of rural residents.

## 1. Introduction

Housing is a place of safety for people. With all of China’s rural poor emerging from poverty by 2020, improving housing quality and the habitat can help enhance the overall wellbeing of rural residents and achieve poverty reduction. Using questionnaire survey data from 2617 peasant households of four poverty-stricken counties in Gansu Province, China, we explored the impact of the Renovation Program of Dilapidated Houses (Wei Fang Gai Zao, hereinafter referred to as RPDH), a major program of Targeted Poverty Alleviation, on peasant households’ satisfaction and the underlying economic logic.

A house is a basic fixed asset and the primary possession of many rural households, and its function has been expanded from simple residential living to productive recreation, which is an important source of subjective wellbeing for rural households [1]. The government carried out the RPDH to give priority to helping the most dilapidated housing and the poorest peasant households to solve the most basic housing safety problems. It provides financial subsidies according to poverty standards through voluntary applications from peasant households and adopts local renovation and nearby reconstruction after government review and approval. At the same time, it focuses on strengthening the design of modern farmhouses, improving the use function of farmhouses, continuously improving the living quality of peasant households, and realizing the sustainability of the human living environment. With the implementation of the Rural Revitalization strategy, there will be large-scale new rural housing construction in China in the coming decades through the implementation of the RPDH [2].

Despite poor supervision, improper use of funds, and lack of equity in the implementation of the RPDH [3], the program has still produced great benefits for participating peasant households. First, those farmer households that are poor due to disability, disease, or disaster and own low economic income have lived in dilapidated and old houses for many years, which seriously threatens their life safety and property security. Implementing the RPDH not only solves the problem of farmers’ housing safety but also provides policy support for farmers to achieve the guarantee of residential rights [4]. Second, with relevant construction technology support, the quality and safety of farmers’ houses are significantly improved, and the problems of water, electricity supply, sewage, and garbage in the village are solved at the same time. In the process of building an environmentally friendly and ecologically civilized countryside combined with the RPDH, the living environment has been improved, and the comfort and satisfaction of farmers’ living has been enhanced [5]. We also provide relevant evidence that the RPDH has significantly improved the living conditions of peasant households. Third, as the reconstruction and renovation of farmers’ houses requires the employment of a large number of individual rural builders and porters, farmers’ employment opportunities have also increased significantly through the development of rural construction and transportation industries. Through various forms of technical training, the RPDH helps surplus rural laborers achieve employment and promotes urban–rural integration [6]. Our evidence also shows that the RPDH has increased the salary income of peasant households. Finally, we demonstrate that because housing also serves a productive function, improving the housing conditions of farm households through government assistance has increased their operating income.

Life satisfaction is a subjective evaluation and indicator of the quality of life based on criteria established by individuals. Numerous studies have found that improving housing conditions can enhance the quality of life and happiness of residents [7,8,9], and housing satisfaction is significantly related to residents’ psychological health and quality of life. Residential housing satisfaction is mainly influenced by housing quality, the surrounding environment, and supporting facilities [10]. It has been found that objective quality of life explains only 15% of residents’ quality of life, whereas subjective indicators such as health status, interpersonal relationships, and life satisfaction explain 70% to 80% [11]. Therefore, subjective score ratings obtained through surveys can effectively reflect the true quality of life and subjective satisfaction [12], which are comparable across different trait groups or classes [13]. By investigating the factors associated with life satisfaction among Chinese older adults living in rural areas, Li et al. [14] found that 60.2% of rural older adults rated their life satisfaction as high, which was significantly related to education, economic resources, self-rated health, and living environment. In addition, annual household income, area of residence, and perceptions of social equity also had significant effects on life satisfaction among rural residents [15]. However, there are few studies on the life satisfaction of rural households in poverty-stricken areas, probably due to the lack of survey data on housing status or questionable indicators used to measure satisfaction. Due to poor economic conditions, are there any differences in perceptions of how the living environment affects life satisfaction? Field research can further clarify the relationship between the two and, thus, facilitate policy evaluation.

In this paper, we combine several unique sources of information to provide a rich set of qualitative and quantitative evidence on satisfaction with the RPDH from the perspective of peasant households. To understand the role of the program in poverty alleviation, we use household questionnaires, defining life satisfaction as “the overall evaluation of one’s quality of life according to one’s own criteria” [16], i.e., the subjective satisfaction that residents receive from living in a particular place. We designed a cross-sectional survey of 2617 peasant households in 57 townships and 142 administrative villages of four poverty-stricken counties in Gansu Province. To reliably estimate the impact of a single policy on dilapidated housing and to exclude potential selection bias as much as possible, we use the Propensity Score Matching (PSM) method to match the treatment group with the control group and pass the balance and co-support tests. Through the study of these survey data, it is found that those peasant households who participate in the RPDH have significantly improved their living conditions compared with those who do not participate in the policy, which verifies our conjecture.

In addition to generating a positive average disposition effect, we also find significant heterogeneity effects. First, we find that the degree of improvement in living conditions of peasant households in non-poverty-stricken villages is significantly higher than that in poverty-stricken villages due to the RPDH. This heterogeneity can be explained by the fact that non-poverty-stricken villages have certain advantages in terms of both hard power and soft power. Since the RPDH is mainly self-financed and supplemented by government subsidies, farm households in non-poverty-stricken villages are more capable of participating in the project, and the satisfaction they have received from it will be higher. Second, the average disposition effect on general-assurance households is higher than that on households with minimal assurance and five assurance. Since minimal-assurance households and five-assurance households are poorer, they often lack labor skills, cannot make ends meet, or are heavily in debt; in these cases, it is difficult for them to come up with the required funds, and the utility of repairing houses is relatively weak.

We further study the mechanism of the impact of the RPDH on farmers’ life satisfaction. We find that the RPDH has a significant impact on farmers’ life satisfaction by increasing per capita wage income, per capita operating income, and per capita net income. This may be explained by the fact that the renovation of dilapidated houses widens farmers’ employment channels, boosts domestic demand, and creates conditions for farmers’ individual business projects, thus increasing their satisfaction.

This study is related to the broad branch of literature that focuses on global anti-poverty practices [17,18,19]. Loayza and Raddatz [20] analyzed the impact of growth in different sectors on poverty reduction through cross-country empirical evidence and found that unskilled labor-intensive sectors (agriculture, construction, and manufacturing) make the largest contribution. The transfer of labor not only increases the per capita living standards of the remaining rural population and optimizes the human–environment relationship but also helps individuals to escape poverty and to achieve stable employment [21]. In addition, we make some contributions to the literature studying the effects of poverty alleviation policies. First, using primary research data from the project team’s trip to Gansu Province, China, we apply the PSM to construct a counterfactual basis for estimating the average disposition effect of participating in the RPDH on the life satisfaction of peasant households, correcting for selectivity bias and controlling for the effects of other policies, and the mechanisms involved are analyzed. Second, we examine the impact of the RPDH from the perspectives of both village attributes and household attributes and explore the heterogeneous effects of the policy, further enriching the empirical studies related to the RPDH. Third, we provide new ideas and perspectives for improving the overall wellbeing of rural residents, achieving sustainability of rural habitats, consolidating the achievements of poverty alleviation, and articulating the policy of RPDH in the context of rural revitalization in China.

The remainder of the paper is organized as follows. Section 2 introduces the RPDH in China. Section 3 presents the research design, including descriptive analysis and research methodology. Section 4 presents the empirical analysis. We first estimate the policy impact and then estimate heterogeneous effects across village attributes and household attributes. In Section 5, we discuss possible impact mechanisms. Section 6 concludes the study.

## 2. Renovation Program of Dilapidated Houses in China

China’s dilapidated houses renovation program was first launched in 2008 with a pilot project in Guizhou Province. In 2009, the Chinese government gradually expanded the pilot projects for the renovation of dilapidated houses in rural areas, including land border counties, counties in ethnic autonomous areas in the western region, and borderline regiments of the Xinjiang Production and Construction Corps. From 2008 to 2012, China arranged a total of CNY 68.172 billion in subsidies for the RPDH, supporting 9.734 million poor farmers to renovate their dilapidated houses, and the pilot projects have gradually covered rural areas nationwide.

In 2012, the Chinese government put forward that by 2020, the poverty alleviation targets would not worry about food or clothing, and their housing security, compulsory education, and basic medical care would be guaranteed. Since then, the renovation of dilapidated houses in rural China has entered a new stage. From 2012 to 2016, the average subsidy standard for the RPDH in China was 7500 yuan per household, and the subsidy was increased by CNY 1000 per household in poor areas. In 2017, the average subsidy standard for the RPDH significantly increased from CNY 8500 yuan to CNY 14,000. In 2019, the Chinese government continued to increase the subsidy standard for the renovation of dilapidated houses. From 2012 to 2020, the central government invested a total of CNY 207.7 billion in subsidies to support a total of 18.65 million poor peasant households in renovating their dilapidated houses, helping more than 60 million poor people leave their original dilapidated mud and straw houses, adobe houses, and other run-down houses and move to safe housing.

### 2.1. Application and Screening

Dilapidated houses refer to houses whose structures have been seriously damaged or whose load-bearing components have been weakened and may lose structural stability and bearing capacity at any time. Thus, the safety of the structures for living purposes cannot be guaranteed. In 2009, the Chinese government announced that the pilot subsidy targets to renovate dilapidated houses in rural areas would focus on five-assurance households, minimal-assurance households (households enjoying the minimum living guarantee), and other poor rural households living in dilapidated houses. According to the risk and damage degree of the house, the houses were divided into four grades: A, B, C, and D. Dilapidated houses are those identified as completely dilapidated (grade D) or partially dilapidated (grade C). Compared with urban areas, the number of people living in dilapidated houses in rural areas is greater, and the degree of damage to and risk from the houses is more serious.

Therefore, the accurate identification of the state of the houses is a crucial first step in the reconstruction of dilapidated houses in rural areas. The Chinese government stipulates that the target of rural RPDH refers to rural five-assurance households, minimal-assurance households, impoverished disabled families, and other impoverished households living in dilapidated houses. Priority is given to helping the poorest farmers living in dilapidated houses to solve the most basic problem of securing safe housing.

Initially, farmers who meet the conditions for the RPDH in rural areas voluntarily submit a written application to the village committee. After the village committee receives the application to renovate the farmer’s dilapidated house, it holds a village meeting to review and publicize it. If there is any question about the review or public announcement, local officials need to carefully review the basic conditions of these applicants to ensure the validity of the list. Then, the town government conducts household surveys, neighborhood visits, and letter requests for the application materials submitted by the village committee to investigate and verify the applicant’s housing and family economic conditions in a timely manner. Finally, the county-level government conducts an on-site review, approves the applications that meet the conditions of the subsidy, and approves the reconstruction funding method and standard according to the appraisal of professionals about the degree of danger of the house in question; the approval results are then made public. Those who do not meet the conditions for the subsidy will not be approved, and the reasons will be explained.

Unlike some local-based poverty alleviation programs, the implementation of the RPDH is determined according to household’s specific situation. During the empirical analysis of this study, we strictly control the household characteristics.

### 2.2. Achievements

**Figure 1:**
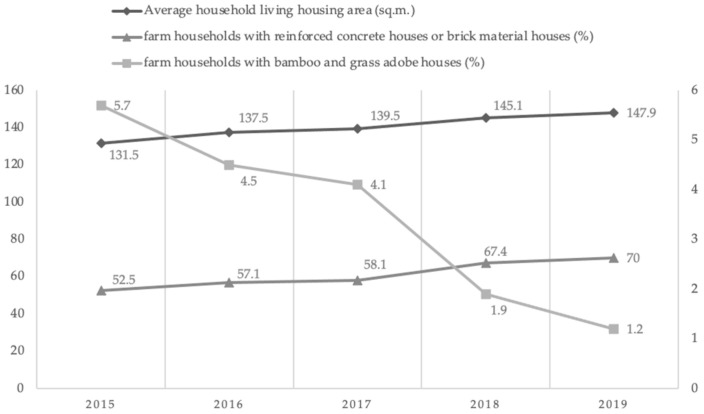
Trends in housing conditions of residents in poor areas in China, 2015–2019. Source: China Rural Poverty Monitoring Report.

The year 2020 is the last year of China’s poverty eradication campaign. As of 2019, the average housing floor area of rural residents in poor areas in China reached 147.9 square meters, an increase of 16 square meters from the average 131.5 square meters per household at the end of 2015. While expanding the housing area of poor households, the Chinese government also promoted the optimization of farm housing structures, with significant improvements in building quality and safety. Many poor households have left their dilapidated bamboo-straw adobe houses with poor facilities and moved into spacious, bright, and well-equipped brick and tile houses. From Figure 1, it can be seen that the proportion of farm households living in bamboo and grass adobe houses in poor areas of China shows an overall decreasing trend, from 5.7% at the end of 2015 to 1.2% at the end of 2019, whereas the proportion of farm households living in reinforced concrete houses or houses made of brick and concrete materials has increased from 52.5% at the end of 2015 to 70.0% at the end of 2019. The number of farm households rebuilt from bamboo and grass adobe houses into brick and concrete structures and those living in reinforced concrete houses or brick and concrete material houses has increased significantly, and the overall housing conditions of farm households in poor areas of China have improved significantly.

**Figure 2:**
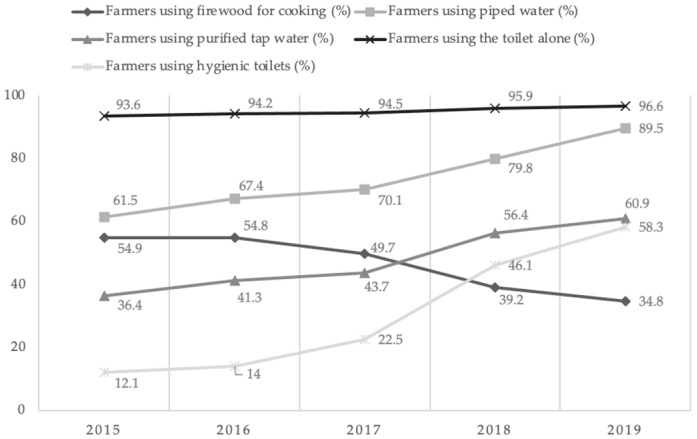
Habitat Improvement Trends in Poverty-stricken Areas in China, 2015–2019. Source: China Rural Poverty Monitoring Report.

At the same time, to improve the living conditions of farmers in poor areas, China has actively helped peasant households introduce natural gas and electricity to replace cooking with firewood that was used in the past. This has largely eliminated the dangers associated with cooking and reduced the negative impact on farmers’ health. As shown in Figure 2, the proportion of households using firewood for cooking has dropped significantly in the past few years, from 54.9% to 34.8%, which has greatly protected the rural environment and contributed to the improvement of rural habitats.

China has also established a unified piped water supply system through scientific treatment. This new system has greatly improved the convenience of drinking water for farmers. The proportion of farmers utilizing well and cellar water has decreased, the proportion of tap water has increased significantly, and the drinking water situation has improved significantly, with an increase of nearly 30 percentage points from 61.5% to 89.5% in the 2015–2019 period. The unified water system has also greatly improved water quality and further ensured the health of farmers’ drinking water. Meanwhile, China has installed suitable drinking water purification and disinfection equipment and established sewage treatment plants based on local conditions. Farmers’ water quality is regularly tested, which has greatly ensured the safety of farmers’ drinking water. As shown in Figure 2, the proportion of farmers using purified tap water increased by more than 20 percentage points from 2015 to 2019, and the safety of farmers’ drinking water further improved, with significant positive effects on farmers’ household infrastructure. In terms of sanitation facilities, the proportion of rural households in poor areas using toilets alone has risen from 93.6% at the end of 2015 to 96.6% in 2019, and the proportion of rural households using sanitary toilets has risen from 12.1% to 46.1%, which shows that China’s “toilet renovation” has achieved remarkable results, and rural residents’ sanitary conditions have improved significantly.

### 2.3. Research

The external environment of policy implementation, such as national strategic goals, the economic development level, and total social demand, will affect the process of policy implementation. As China proposes to build a new countryside, and as it promotes the continuous expansion of poverty alleviation policy practices, the RPDH has gradually become one of the focuses of academic research.

On the one hand, the existing research has summarized the problems and experiences in the implementation process of the RPDH. Based on the Horn–Mitter Model, some scholars have analyzed the factors influencing the implementation of the RPDH in rural areas and found that the out-of-reality policy standards, insufficient policy resources, and the conflict between the government’s macro-strategy and the farmers’ micro-needs have made the implementation of the RPDH difficult [3,22]. In addition, the incomplete information of poor farmers has made it difficult to achieve classified guidance and subsidies, and the different participation willingness of poor farmers has also led to unsatisfactory policy implementation effects [23].

On the other hand, some scholars have used theory and data to test the policy effect of the RPDH. Using the Computable General Equilibrium (CGE) model, Du et al. [24] conducted an in-depth analysis of the transmission mechanism of the RPDH on improving residents’ social welfare and its important impact on economic growth. Taking the example of dangerous house renovation for rural poor families in Hangzhou, Zhejiang Province, Zhu [25] applied a combination of comparative analysis and public opinion surveys in two dimensions, direct performance and driving effect, and found that the policy has achieved significant results in the improvement of living conditions, the reduction of housing burdens, and the innovation of working mechanisms, and at the same time, it has also promoted social development, democratic politics, and ecological civilization.

### 2.4. Other Poverty Alleviation Policies

Since the reform and opening-up, China has successfully emerged from a traditional poverty alleviation and development path, reaching new levels of intensity, breadth, depth, and precision. Since these poor households are usually covered by multiple poverty alleviation policies throughout the sample period, including in the areas of education poverty, medical poverty, industrial poverty, employment poverty, and financial poverty, achieving improved housing is the most direct manifestation of the general public’s sense of wellbeing and security. To clarify the actual effect of the RPDH, we control for the effects of these other poverty alleviation programs (i.e., confounding programs) in our empirical analysis.

## 3. Materials and Methods

### 3.1. Data Description

The data used in this study come from a questionnaire survey on the effectiveness of poverty alleviation in Gansu Province conducted by the China Institute for Poverty Reduction, Beijing Normal University, in 2020. The project team members used a stratified random sampling method to select 57 townships and 142 administrative villages of 4 poverty-stricken counties in Gansu Province for the household survey. Fifty percent of the villages were selected from relatively remote areas with weak foundations, and 50% were randomly sampled within the county. A total of 2635 questionnaires were distributed, of which 2617 were valid, with an efficiency rate of 99.31%. The content of the questionnaire mainly includes the basic situation of family members, access to precise poverty alleviation policies (including housing, education, medical care, industry, employment, and six financial aspects), labor and employment, living conditions, etc. Meanwhile, the respondents’ satisfaction with the current living situation was selected according to “very satisfied, satisfied, relatively satisfied, unsatisfied”. The basic information of the respondents is summarized in Table 1.

### 3.2. Propensity Score Matching

If the life satisfaction of poor farmers can be observed separately in the two cases of those who participate in the RPDH and those who do not, then the difference between the two is the disposition effect of the RPDH. However, in the actual situation, it is difficult to estimate the disposition effect, because the two cases cannot be observed simultaneously. On the one hand, the implementation of the RPDH is not completely exogenous and random; for example, household characteristics are also important influencing factors [26]. On the other hand, precise poverty alleviation policies other than the RPDH also have an impact on the life satisfaction of poor farmers, so it is more difficult to distinguish the causal disposition effects caused by the above factors. The single comparison of the life satisfaction of poor farmers who participated in the RPDH and those who did not participate in this policy would lead to selection bias. Therefore, a counterfactual framework must be constructed if the dispositional effects of the RPDH are to be obtained [27].

To reduce the effects of these biases and confounding variables, we used PSM, a statistical method proposed by Paul Rosenbaum and Donald Rubin [27]. It is assumed that D_i_ = 1 is the treatment group, i.e., peasant households who participate in RPDH, and D_i_ = 0 is the control group, i.e., peasant households who do not participate in the RPDH. Y_1i_ denotes the life satisfaction of the treatment group, and Y_0i_ denotes the life satisfaction of the control group.

First, we calculate the propensity score (P-Score) for each peasant household, i.e., the conditional probability of a peasant household entering the treatment group given the matching variable:

p(x_i_) = P(D_i_ = 1 |x = x_i_) (1)

Then, we match the samples of the treatment group with those of the control group, and the results of the control group samples with the most similar sample characteristics are used as the counterfactual results of the treatment group. To ensure that the matching results are robust, we use the one-to-four nearest neighbor matching method, the caliper matching method, and the kernel matching method to test the findings.

Finally, after the matched samples satisfy the balance and common support tests, we calculate the difference between the means of the treatment and control groups, i.e., the average treatment effects on the treated (ATT):

ATT = E[Y_1i_ |D_i_ = 1, p(x_i_)] − E[Y_0i_ |D_i_ = 0, p(x_i_)] (2)

### 3.3. Variable Description

#### 3.3.1. Outcome Variables

The questionnaire posed the following question: Are you satisfied with the general situation (production and living conditions) of your home since the RPDH? If the answer is “very satisfied”, the value is 3, “satisfied” is 2, “relatively satisfied” is 1, and “unsatisfied” is 0. Table 2 presents the results of all valid questionnaires on life satisfaction, and statistically, it is clear that most of the peasant households are very satisfied with the living conditions of their families, with no unsatisfied answers.

#### 3.3.2. Matching Variables

In this paper, 16 matching variables are selected from four levels, considering the implementation process of China’s poverty alleviation policies and the actual situation of local farmers (Table 3). Regarding the poverty alleviation policy, considering that the outcome variable is farmers’ life satisfaction, and that the improvement of their lives is largely influenced by other precise poverty alleviation policies, education, medical, industrial, employment, and financial poverty alleviation policies are used as matching variables to reduce the interference of other policies. The second issue is the basic situation of the head of the household, who, as the head of the family, has a certain discursive power and is closely related to the household situation. Referring to the selection of household head characteristics variables by Chen et al. [28], three variables are introduced: age, gender, and education level of the head of household. Additionally, we refer to Li et al. [29] for the extension of the Mincer income equation by introducing the variable of the square of the age of the household head. The third is the household demographic structure. Referring to Wang et al. [30], three variables for household characteristics are introduced: the burden of support, the proportion of working individuals, and the number of disabled individuals. Fourth, regarding household economic conditions, the variables include meat-eating frequency, drinking water security, number of owned houses, and per capita net household income. To avoid the effect of the data outline, we make the income category data logarithmic.

Table 4 shows the descriptive statistics of the main variables. There are significant differences in the degree of life satisfaction between the treatment and control groups. The significant differences between the two groups of farmers in terms of whether they enjoy industrial policies, financial policies, the education level of the head of the household, and the proportion of workers indicate that it is essential to control for these individual characteristics.

## 4. Results

Balance tests and common support tests are required before adopting the PSM method to ensure the validity and stability of the method.

### 4.1. Balance Test

**Figure 3:**
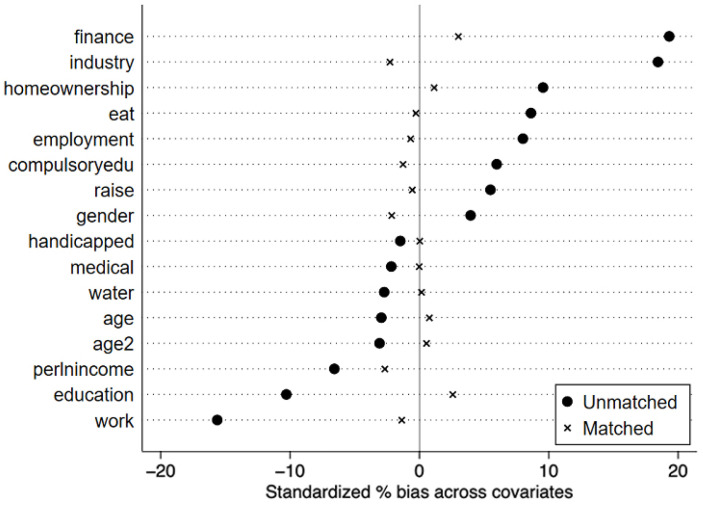
Standardized differences of matching variables before and after matching.

We first perform a balance test on the matched samples, which requires no systematic differences. As shown in Figure 3, the standardized deviations of the matched variables are all significantly reduced and less than 5%. Table 5 illustrates that the mean values of the matched treatment and control samples are similar, and the t test results all accept that there is no systematic difference between the treatment and control groups. Therefore, the matched samples satisfied the overall equilibrium condition.

Table 6 shows the results of the joint test for the balance of variables before and after the matching of samples. The R^2^ of the post-matching regression is small, indicating that whether individuals participate in the RPDH is random for the post-matching sample. The mean standardized difference (B) of the linear index of propensity scores after matching is 7.3, and according to Rubin’s view, B is less than 25, indicating that the variables are balanced. In summary, the matched variables passed the balance test.

### 4.2. Common Support Test

**Figure 4:**
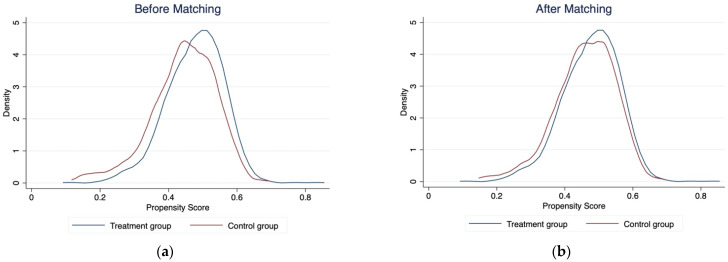
Kernel density plot of propensity scores before and after matching.

The PSM method must satisfy the common support test to ensure its matching quality and efficiency. Table 7 lists the range of common values of propensity score matching samples (only one-to-four nearest neighbor matching method is shown). After matching, only three samples are lost, accounting for 0.11%, indicating that the common support domain is large, and the sample loss has less impact on the regression results. Figure 4a,b shows the kernel density of propensity scores before and after sample matching. The common support domain becomes larger after matching, differences between groups become smaller, and the estimation results are reliable.

### 4.3. Regression Results Analysis

After passing the balance test and the common support test, the average disposition effect of the impact of the RPDH on the life satisfaction of poor farmers is estimated based on the PSM. To avoid the fact that the reported standard errors do not take into account the propensity scores as estimates and to relax the homoscedasticity assumption, we use the Bootstrap method and repeat the sampling 500 times to obtain the standard errors. As seen from Table 8, the ATT estimated by the three methods of one-to-four nearest neighbor matching, caliper matching, and kernel matching are 7.4%, 8%, and 7.7%, respectively, and all are significant at the 1% confidence level, indicating that the results are consistent, and significance does not change based on the choice of matching method. The regression results indicate that there are significant differences in satisfaction between the treatment and control groups, and the RPDH significantly improves the life satisfaction of poor peasant households and increases their sense of wellbeing.

### 4.4. Heterogeneity Analysis

#### 4.4.1. Village Attributes

Village attributes differ, and the improvement effects of the RPDH may vary cross villages. Among the 142 administrative villages in our field research, there are 56 poverty-stricken villages and 86 non-poverty-stricken villages. Compared with poverty-stricken villages, non-poverty-stricken villages have certain advantages in both hard and soft strengths. Although there is a uniform minimum construction standard in the renovation of dilapidated houses, the influence of village infrastructure, overall construction style, and technical level of the construction team may lead to different experiences of poor farmers. To verify the differences caused by the village attributes, we further subsample the regressions. Table 9 presents the average disposition effects for poverty-stricken and non-poverty-stricken villages under the three propensity matching methods. We find that the average disposition effect is higher for non-poverty-stricken villages than for poverty-stricken villages, which by the three methods of one-to-four nearest neighbor matching, caliper matching, and kernel matching are 9.6%, 9.8% and 11.2%, respectively. However, this effect is no longer significant for poverty-stricken villages. It indicates that the impact of the RPDH on the life satisfaction of farm households in non-poverty-stricken villages is greater.

#### 4.4.2. Household Attributes

In terms of household attributes, our survey samples include general-assurance households, minimal-assurance households, and five-assurance households (poverty levels increase in order). Compared with general-assurance households, minimal-assurance households and five-assurance households have lower per capita net household income, less labor force, fewer economic sources, and more difficult living conditions, and their livelihoods tend to be more dependent on the payment of monthly low-income and five-guarantee payments and other government income transfers. The actual effect on the renovation of dilapidated houses may be different from that of general-assurance households. We further divide the sample into minimal-assurance households and five-assurance households versus general-assurance households. As seen from Table 10, the average disposition effects of both sample groups are significantly positive under all three propensity score matching methods, and the general-assurance households are significantly higher than the minimal-assurance households and five-assurance households, indicating that the impact of the dilapidated house renovation policy on the life satisfaction of the better-off farm households is greater. This may be because people with poor living conditions are more dependent on income, and the average disposition effect of repairing or rebuilding houses is relatively weaker from their subjective perspective.

## 5. Discussion

It is generally believed that the higher the level of income, the greater the happiness of residents. This study found that the precise poverty alleviation policy was not only a kind of “social assistance” but also had a facilitating effect on the income growth of farm households [26].

First, the RPDH has increased the wage income of farmer households to a certain extent. Due to the huge scale of rural dilapidated housing renovation and the low degree of mechanization of rural housing construction, the labor-intensive nature of the RPDH is significant. Not only do some farmers carry out the construction themselves, but the RPDH also employs a large number of rural construction craftsmen and temporary workers, which provides abundant jobs in rural areas. These jobs involve construction machinery processing, decoration, utility renovation industry, and storage- and transportation-related services, which largely broaden the employment of rural workers, increase their income, and enhance their life satisfaction. For example, there are many remote villages in the central and western regions of Gansu Province, and the construction materials needed for the renovation of dilapidated houses in rural areas require much long-distance transportation, which greatly promotes the development of the transportation industry and its employment. Gansu Province has also carried out various forms of vocational and technical training to help returning migrant workers and rural laborers who are interested in employment opportunities in village construction. Gansu Province has set up a pool of technical service experts of more than 300 professional and technical personnel to provide field guidance services in batches to various regions throughout the province to provide vocational and technical training classes for migrant workers to obtain a high wage income. At the same time, a large number of jobs motivates workers who have left their hometowns to return, promoting the transfer of labor from towns to rural areas, providing manpower support for rural construction, greatly alleviating rural labor shortages, promoting the comprehensive and sustainable development of rural areas, and improving the happiness of farmers.

Second, the RPDH also improves the operating income of farm households. Farmers’ requirements for housing often have both residential and productive functions. Generally, houses should be built with two or more floors so that some crops, such as grains, can be dried on the first floor, and wide houses can greatly enhance the production efficiency of crops. At the same time, courtyards with more space help farmers carry out farming activities involving animals, such as chickens, ducks, and sheep, which broaden the income channels of farmers and increase their operating income, thus improving their life satisfaction. Therefore, it can be considered that the RPDH is an endogenous development driven by exogenous sources [32], which narrows the urban–rural gap and eventually achieves common prosperity by stimulating endogenous power in poor areas [33].

We refer to the previous research methods on the happiness of residents, since the dependent variable of farmers’ life satisfaction in the sample is discrete data characterized by classification, not continuous information, i.e., “unsatisfied = 0 (not present in the final sample), relatively satisfied = 1, satisfied = 2, very satisfied = 3”, and this group of data has a natural ranking. The higher the level of satisfaction, the better. Therefore, we first use the traditional Ordered Logit model for estimation. However, the traditional ordered logit model has some limitations; that is, the model sets the parameter to a fixed value that does not change with the variation in individual life satisfaction. However, in real life, farmers may have different feelings about the impact of their income on life satisfaction. For example, some may think that material income is not important and instead pursue spiritual goals; others have old and young children, and their burden of living is heavier, so the improvement of living conditions and direct increases in income may have a greater impact on happiness. Therefore, we also introduce the Generalized Ordered Logit model [34], which considers the heterogeneous effects of the independent variables on the explanatory variables at different thresholds, i.e., it relaxes the assumption of parallelism. We assume that the coefficients of the independent variables may change between different satisfaction levels.

(3) $$Y_{i}^{*}=\alpha {Χ}_{i}+\varepsilon_{i}\;,\;i=1,2,3$$

(4) $$Y_{i}=\{\begin{array}{l} 1,\;\;\;\;\;\;\;\;\;Y_{i}^{*}\leq \beta_{1}{Χ}_{i}\\ 2,\;\;\beta_{1}{Χ}_{i}<Y_{i}^{*}\leq \beta_{2}{Χ}_{i}\\ 3,\;\;\;\;\;\;\;\;\;Y_{i}^{*}>\beta_{2}{Χ}_{i} \end{array}$$

where $Y_{i}^{*}$ is the unobservable latent variable, $Y_{i}$ is the observable variable of peasant households’ life satisfaction, including 1, 2, and 3, and ${Χ}_{i}$ denotes the control variable. The impact of income change caused by the renovation of dilapidated houses varies with the change in life satisfaction. When farmers’ life satisfaction changes from “1” to “2”, it depends on $\beta_{1}X$. When it changes from “2” to “3”, it depends on $\beta_{2}X$. The cumulative distribution function of the random disturbance term $\varepsilon_{i}$ is assumed to be Ψ(x), and the probabilities of farmers’ life satisfaction $Y_{i}=1,\;2,\;3$ are as follows:

(5) $$P\left(Y_{i}=1|X_{i}\right)=P\left(\alpha {Χ}_{i}+\varepsilon_{i}\leq \beta_{1}{Χ}_{i}|{Χ}_{i}\right)=\Psi \left(\beta_{1}{Χ}_{i}-\alpha {Χ}_{i}\right)$$

(6) $$P\left(Y_{i}=2|X_{i}\right)=P\left(\beta_{1}{Χ}_{i}<\alpha {Χ}_{i}+\varepsilon_{i}\leq \beta_{2}{Χ}_{i}|{Χ}_{i}\right)=\Psi \left(\beta_{2}{Χ}_{i}-\alpha {Χ}_{i}\right)-\Psi \left(\beta_{1}{Χ}_{i}-\alpha {Χ}_{i}\right)$$

(7) $$P\left(Y_{i}=3|X_{i}\right)=P\left(\alpha {Χ}_{i}+\varepsilon_{i}>\beta_{2}{Χ}_{i}\right)=1-\Psi \left(\beta_{2}{Χ}_{i}-\alpha {Χ}_{i}\right)$$

When the independent variable takes the value of $x_{k}$, the corresponding marginal effect of the peasant household’s life satisfaction is calculated as follows:

(8) $$\frac{\partial E[y|x]}{\partial x}{|}_{x=x_{k}}=\frac{\partial F\left[x\beta \right]}{\partial x}{|}_{x=x_{k}}=f\left(x_{k}\beta \right)\beta$$

We conducted Generalized Ordered Logit regressions and traditional Ordered Logit regressions on peasant households’ life satisfaction with the interaction terms of the RPDH and logarithm of wage income, operating income, and net per capita household income to investigate how the RPDH affects peasant households’ life satisfaction through income levels.

From left to right, Table 11 shows the regression results of the Generalized Ordered Logit model with different threshold heterogeneity coefficients and the regression results of the traditional Ordered Logit model. The comparison shows that the regression results of both are basically consistent, indicating that the increase in income level driven by the RPDH promotes the improvement of farmers’ life happiness. The regression results of the Generalized Ordered Logit model indicate that this enhancement effect is more significant among those who have higher levels of life satisfaction; in contrast, for those who are not so satisfied with their lives, the increase in income because of the RPDH has little effect. Then, we conduct a parallelism test on the traditional Ordered Logit model and find that the original hypothesis is rejected at the 1% significance level, so the Generalized Ordered Logit model is finally established.

Furthermore, Table 12 shows the marginal effects of each explanatory variable for different thresholds of life satisfaction under the Generalized Ordered Logit model (we only show the interaction terms due to space limitations).

From the perspective of the size of marginal effect, the changes in operating income, wage income, and per capita net income caused by the RPDH have basically the same impact on the life satisfaction of farmers. That is, as the income level of farmers who own dilapidated houses improves, the probability of low life satisfaction of farmers (y = 1, 2) begins to decrease, and the probability of a higher level of satisfaction (y = 3) increases. Therefore, the assumption that the RPDH improves the income of poor rural residents and eventually enhances happiness and satisfaction is verified.

## 6. Conclusions

The Renovation Program of Dilapidated Houses in rural areas is an important policy that benefits rural residents, supports agriculture, and helps the poor. It is a systematic project launched by the Chinese government to improve the housing conditions of the rural poor, ensure the realization of housing rights, and promote the construction of new rural areas and sustainable social and economic development. As an important policy measure in response to the goal of “meeting the housing needs of all people”, the RPDH is of great significance for improving people’s livelihoods. Members of our research team visited four counties in Gansu Province, China, and used the first-hand data of household surveys to conduct an empirical analysis on the effect of farmers’ subjective evaluation of dilapidated house renovation projects and discussed the possible impact mechanism.

The results of the study find that, regardless of the PSM matching method used, the RPDH has led to a significant increase in life satisfaction among poor peasant households, and this average disposition effect is higher among non-poverty-stricken villages and general-assurance households. The empirical results all pass the equilibrium and co-support tests, and the findings are robust and reliable. In further mechanism analysis, Generalized Ordered Logit model regression is used to find that the RPDH improves the per capita net income level and life satisfaction of peasant households by increasing their wage income and operating income, which is more effective in highly satisfied people.

There are more research topics worth exploring in the future. First, the policy of renovating dilapidated houses is only one of China’s poverty alleviation initiatives. The actual effects of different poverty alleviation programs can be synthesized to determine the most effective combination to improve the life satisfaction of farm households in the rural revitalization stage. Second, due to research time constraints and data acquisition, we only studied one-year data in the four counties of Gansu Province. It would be worthwhile to further investigate the regional and temporal heterogeneity of dilapidated house renovation policies once more regional data and site observations become available.

## Figures and Tables

**Table 1:** Basic information of respondents.

	Category	Proportion (%)
Gender	Male	92.71
	Female	7.29
Age	<30	0.88
	30–39	7.26
	40–49	19.18
	50–59	40.47
	>60	32.31
Education Background	Primary school and below	54.15
	Junior high school	40.92
	High school	4.7
	Vocational school or technical secondary school	0.08
	Junior college or above	0.15
Number of persons with disabilities	0	80.43
	1	16.13
	≥2	3.44
Burden of raising	<25%	46.24
	25–50%	31.52
	>50%	22.24
Number of houses owned	0	3.06
	1	96.03
	≥2	0.91
Net household income per capita ^1^	<6000	17.99
	6000–10,000	46.39
	10,000–15,000	22.43
	>15,000	13.19
Participate in RPDH	Yes	45.89
	No	54.11

^1^ In CNY.

**Table 2:** Life satisfaction of peasant households with valid questionnaires.

	Category	Proportion (%)
Satisfaction	Very satisfied	73.48
	Satisfied	26.09
	Relatively satisfied	0.43
	Unsatisfied	0

**Table 3:** Definition of the main variables.

Variable Name	Specific Definition
satisfaction *	“Very satisfied” = 3; “Satisfied” = 2; “Relatively satisfied” = 1.
Compulsoryedu ^1^	Education poverty alleviation policy means that students in the compulsory education stage at home enjoy education poverty alleviation policy in at least one of the following ways: yes = 1, no = 0. ① Free nutritious meals; ② boarding subsidies; ③ free of tuition and miscellaneous fees; ④ free of book fees.
medical ^1^	Medical poverty alleviation policy means that patients with serious diseases or chronic diseases in the family can enjoy medical poverty alleviation policy in at least one of the following ways: yes = 1, no = 0. ① If there are long-term chronic patients, do they enjoy services from a family doctor? ② If there are serious patients, do they go to the hospital to see a doctor and enjoy deposit-free, one-stop reimbursement and other services?
industry ^1^	Farmers enjoy the industrial poverty alleviation policy in at least one of the following ways [31]: yes = 1, no = 0. ① Develop the industry independently with the help of funds, physical materials, or technical support provided by the government. ② Buy shares in cooperatives. ③ Develop the industry under the leadership of enterprises, cooperatives, and large households.
employment ^1^	The employment poverty alleviation policy includes the following three aspects, and if farmers enjoy at least one aspect, they belong to the employment poverty alleviation policy: yes = 1, no = 0. ① Does anyone in the family participate in job training? ② Does the family arrange for migrant work through the government? ③ Does anyone in the family participate in public welfare posts or poverty alleviation workshops?
finance ^1^	Financial poverty alleviation policy mainly refers to small loans for poverty alleviation, which is marked as 1 if the family has borrowed small loans for poverty alleviation and 0 otherwise.
age ^2^	The householders’ ages.
gender ^2^	male = 1, female = 0.
education ^2^	Primary school and below = 1, middle school = 2, high school = 3, vocational school, technical secondary school = 4, junior college, and above = 5.
handicapped ^3^	Number of disabled individuals.
raise ^3^	Burden of raising a family: proportion of children under 16 years old and people over 60 years old in total population.
work ^3^	Migrant worker proportion: proportion of people engaged in nonagricultural industries of total household population.
eat ^4^	Meat frequency: eat meat whenever you want = 5; eat meat every third meal (no less than once a week) = 4; sometimes eat meat (no less than once a month) = 3; eat meat only on holidays = 2; never eat meat because you cannot afford it = 1; never eat meat = 0 for noneconomic reasons such as living habits.
water ^4^	Drinking water safety: perennial safety of water quality = 3; unsafe water quality for no more than 1 month throughout the year = 2; annual unsafe water quality for more than 1 month = 1; I do not know = 0.
homeownership ^4^	Number of houses owned.
lnperincome ^4^	The logarithm of household net income per capita.

^1^ Targeted poverty alleviation policies. ^2^ Basic information of household heads. ^3^ Family demographic structure. ^4^ Family economic conditions. * All these are matching variables except satisfaction.

**Table 4:** Descriptive statistics of the main variables.

Variable Name	All the Samples			Treatment Group	Control Group	Difference of Significance
	Min	Max	Mean (1)	Mean (2)	Mean (3)	(2) − (3) = (4)
Outcome variables						
satisfaction	1	3	2.731 (0.453)	2.774 (0.422)	2.694 (0.475)	0.080 *** (0.177)
Matching variables						
compulsoryedu	0	1	0.322 (0.467)	0.337 (0.473)	0.309 (0.462)	0.028 (0.018)
medical	0	1	0.423 (0.494)	0.417 (0.493)	0.428 (0.495)	−0.011 (0.019)
industry	0	1	0.964 (0.185)	0.983 (0.131)	0.949 (0.220)	0.034 *** (0.007)
employment	0	1	0.730 (0.444)	0.749 (0.434)	0.714 (0.452)	0.035 * (0.017)
finance	0	1	0.702 (0.458)	0.749 (0.434)	0.662 (0.473)	0.087 *** (0.018)
age	22	90	55.330 (11.003)	55.154 (10.942)	55.480 (11.056)	−0.326 (0.432)
gender	0	1	0.927 (0.260)	0.933 (0.251)	0.922 (0.268)	0.011 (0.102)
education	1	5	1.512 (0.606)	1.478 (0.601)	1.540 (0.608)	−0.062 ** (0.024)
handicapped	0	3	0.233 (0.510)	0.229 (0.516)	0.237 (0.504)	0.008 (0.020)
raise	0	100	34.060 (30.319)	34.960 (30.641)	33.296 (30.034)	1.664 (1.189)
work	0	100	21.821 (21.911)	19.975 (21.139)	23.387 (22.433)	3.412 *** (0.857)
eat	0	5	4.606 (0.775)	4.642 (0.726)	4.576 (0.813)	0.066 ** (0.030)
water	0	3	2.989 (0.150)	2.987 (0.168)	2.991 (0.132)	−0.004 (0.006)
homeownership	0	4	1.037 (0.223)	1.048 (0.244)	1.027 (0.204)	0.021 ** (0.009)
lnperincome	8.118	11.142	9.101 (0.446)	9.086 (0.422)	9.115 (0.465)	−0.029 * (0.018)
N	2617			1201	1416	

***, **, and * indicate significance at the levels of 1%, 5%, and 10%, respectively. Columns (1), (2), (3) are the standard deviation in parentheses, and column (4) is the standard error. All results are reserved to three decimal places, the same below.

**Table 5:** Balance test results.

Variable Name		Mean Difference Test				Standardized Test	
	Unmatched Matched	Treatment Group	Control Group	*t* Test	*p* Value	Standardized Differences	Drop (%)
compulsoryedu	U	0.3072	0.3093	1.52	0.128	6.0	78.3
	M	0.3072	0.3433	−0.31	0.775	−1.3	
medical	U	0.4172	0.428	−0.56	0.577	−2.2	98.1
	M	0.4165	0.4167	−0.01	0.992	0.0	
industry	U	0.9825	0.9492	4.61	0.000	18.4	87.5
	M	0.9833	0.9875	−0.85	0.395	−2.3	
employment	U	0.7494	0.714	2.03	0.042	8.0	91.2
	M	0.7496	0.7527	−0.18	0.859	−0.7	
finance	U	0.7494	0.6617	4.91	0.000	19.3	84.5
	M	0.7496	0.736	0.76	0.448	3.0	
age	U	55.154	55.48	−0.75	0.451	−3.0	74.8
	M	55.174	55.092	0.18	0.855	0.7	
age^2^	U	3161.6	3200.1	−0.79	0.430	−3.1	83.1
	M	3163.8	3157.3	0.13	0.897	0.5	
gender	U	0.9326	0.9223	1.00	0.316	3.9	45.0
	M	0.9324	0.938	−0.56	0.576	−2.2	
education	U	1.4779	1.5403	−2.63	0.009	−10.3	75.2
	M	1.4783	1.4629	0.64	0.521	2.6	
handicapped	U	0.229	0.2366	−0.38	0.704	−1.5	100.0
	M	0.2296	0.2296	0.00	1.000	0.0	
raise	U	34.959	33.296	1.40	0.162	5.5	89.5
	M	34.993	35.168	−0.14	0.887	−0.6	
work	U	19.975	23.387	−3.98	0.000	−15.7	91.1
	M	19.925	20.229	−0.35	0.725	−1.4	
eat	U	4.642	4.5756	2.19	0.029	8.6	96.5
	M	4.6419	4.6442	−0.08	0.938	−0.3	
water	U	2.9867	2.9908	−0.7	0.481	−2.7	95.0
	M	2.9866	2.9864	0.03	0.977	0.1	
homeownership	U	1.0483	1.0268	2.45	0.014	9.5	88.3
	M	1.0434	1.0409	0.27	0.789	1.1	
perlnincome	U	9.0856	9.1148	−1.67	0.094	−6.6	59.1
	M	9.0838	9.0958	−0.67	0.503	−2.7	

**Table 6:** Results of the joint test of the balance of matching variables before and after matching.

Sample	Ps R^2^	LR chi2	MeanBias	MedBias	B
Unmatched	0.025	90.73	7.8	6.3	37.5
Matched	0.001	3.19	1.2	0.9	7.3

**Table 7:** The propensity score matches the common value range of samples.

Matched Sample Classification	Outside	Within	All the Samples
Treatment group	3	1198	1201
Control group	0	1416	1416
All the samples	3	2614	2617

**Table 8:** The ATT of the Renovation Program of Dilapidated Houses.

	Nearest Neighbor Matching (K = 4)	Caliper Matching	Nuclear Matching
ATT	0.074 *** (3.096)	0.080 *** (4.419)	0.077 *** (4.209)
Treatment group	1198	1198	1198
Control group	1416	1390	1410
All the samples	2614	2588	2617

T values are in parentheses. *** indicates significance at the levels of 1%.

**Table 9:** Attribute heterogeneity of villages.

Methods	Category	Poverty-Stricken Village	Non-Poverty-Stricken Village
Nearest neighbor matching (K = 4)	ATT	0.029 (0.771)	0.096 *** (3.073)
	Treatment group	557	644
	Control group	562	834
	All the samples	1119	1478
Caliper matching	ATT	0.034 (0.771)	0.098 *** (3.073)
	Treatment group	542	633
	Control group	559	829
	All the samples	1101	1462
Nuclear matching	ATT	0.038 (1.273)	0.112 *** (4.760)
	Treatment group	556	644
	Control group	562	834
	All the samples	1118	1478

T values are in parentheses. *** indicates significance at the levels of 1%.

**Table 10:** Attribute heterogeneity of households.

Methods	Category	Minimal-Assurance Households and Five-Assurance Households	General-Assurance Households
Nearest neighbor matching (K = 4)	ATT	0.067 * (1.660)	0.093 *** (3.421)
	Treatment group	387	804
	Control group	436	967
	All the samples	823	1771
Caliper matching	ATT	0.064 * (1.660)	0.093 *** (3.421)
	Treatment group	386	804
	Control group	432	944
	All the samples	818	1748
Nuclear matching	ATT	0.075 ** (2.360)	0.077 *** (3.576)
	Treatment group	387	804
	Control group	436	961
	All the samples	823	1765

T values are in parentheses. ***, **, and * indicate significance at the levels of 1%, 5%, and 10%, respectively.

**Table 11:** Regression results of mechanisms.

	Generalized Ordered Logit Model						Traditional Ordered Logit Model		
Variables	$\beta_{1}$	$\beta_{2}$	$\beta_{1}$	$\beta_{2}$	$\beta_{1}$	$\beta_{2}$			
RPDH × lnoperate	0.149	0.043 ***					0.043 ***		
	(1.48)	(3.86)					(3.89)		
RPDH × lnsalary			0.139	0.033 ***				0.033 ***	
			(1.55)	(3.35)				(3.40)	
RPDH × lnperincome					0.174 *	0.043 ***			0.043 ***
					(1.82)	(4.18)			(4.23)
compulsory-edu	−0.948	−0.050	−0.918	−0.079	−0.920	−0.044	−0.053	−0.082	−0.047
	(−1.27)	(−0.47)	(−1.23)	(−0.74)	(−1.23)	(−0.41)	(−0.50)	(−0.77)	(−0.44)
medical	0.086	−0.001	0.101	−0.004	0.146	−0.002	−0.003	−0.005	−0.003
	(0.12)	(−0.01)	(0.14)	(−0.04)	(0.21)	(−0.02)	(−0.03)	(−0.05)	(−0.04)
industry	2.364 **	−0.489 *	2.266 **	−0.472 *	2.292 **	−0.504 *	−0.451 *	−0.433 *	−0.463 *
	(2.53)	(−1.86)	(2.41)	(−1.80)	(2.45)	(−1.91)	(−1.72)	(−1.65)	(−1.76)
employment	0.783	0.354 ***	0.732	0.345 ***	0.735	0.363 ***	0.358 ***	0.348 ***	0.367 ***
	(1.23)	(3.34)	(1.12)	(3.25)	(1.13)	(3.43)	(3.38)	(3.29)	(3.47)
finance	−0.155	−0.059	−0.117	−0.047	−0.186	−0.058	−0.061	−0.048	−0.060
	(−0.22)	(−0.58)	(−0.17)	(−0.46)	(−0.27)	(−0.57)	(−0.60)	(−0.48)	(−0.59)
age	−0.267	0.071 **	−0.252	0.078 **	−0.257	0.077 **	0.070 **	0.077 **	0.076 **
	(−0.83)	(2.27)	(−0.81)	(2.51)	(−0.82)	(2.49)	(2.24)	(2.48)	(2.46)
age2	0.003	−0.001 **	0.003	−0.001 **	0.003	−0.001 **	−0.001 **	−0.001 **	−0.001 **
	(0.96)	(−2.33)	(0.97)	(−2.55)	(0.97)	(−2.54)	(−2.29)	(−2.52)	(−2.51)
gender	0.699	−0.137	0.555	−0.128	0.796	−0.114	−0.133	−0.125	−0.110
	(0.59)	(−0.77)	(0.47)	(−0.72)	(0.68)	(−0.65)	(−0.75)	(−0.71)	(−0.62)
education	−0.672	−0.087	−0.710	−0.089	−0.716	−0.086	−0.088	−0.090	−0.087
	(−1.21)	(−1.13)	(−1.28)	(−1.16)	(−1.28)	(−1.11)	(−1.14)	(−1.17)	(−1.12)
handicapped	−0.313	−0.117	−0.295	−0.110	−0.368	−0.117	−0.117	−0.110	−0.117
	(−0.52)	(−1.35)	(−0.50)	(−1.27)	(−0.6 2)	(−1.34)	(−1.35)	(−1.27)	(−1.35)
raise	−0.001	0.004 *	−0.000	0.005 **	−0.001	0.004 *	0.004 **	0.005 **	0.004 *
	(−0.03)	(1.96)	(−0.02)	(2.18)	(−0.06)	(1.93)	(1.96)	(2.19)	(1.94)
work	−0.010	0.001	−0.014	−0.001	−0.011	0.000	0.001	−0.001	0.000
	(−0.68)	(0.29)	(−0.90)	(−0.24)	(−0.72)	(0.19)	(0.25)	(−0.29)	(0.15)
eat	0.531 *	0.261 ***	0.524 *	0.266 ***	0.521 *	0.264 ***	0.266 ***	0.271 ***	0.269 ***
	(1.88)	(4.78)	(1.86)	(4.87)	(1.84)	(4.84)	(4.88)	(4.97)	(4.93)
water	−5.959	0.393	−4.685	0.433	−4.676	0.431	0.385	0.425	0.423
	(−0.01)	(1.47)	(−0.01)	(1.61)	(−0.01)	(1.60)	(1.46)	(1.61)	(1.59)
homeowner-ship	−2.989 ***	0.300	−3.037 ***	0.312	−3.080 ***	0.307	0.249	0.261	0.255
	(−3.58)	(1.32)	(−3.55)	(1.37)	(−3.60)	(1.35)	(1.11)	(1.15)	(1.13)
Constant	27.823	−3.185 **	23.865	−3.519 ***	23.716	−3.546 ***	—	—	—
	(0.02)	(−2.51)	(0.02)	(−2.78)	(0.02)	(−2.79)			
N	2617	2617	2617	2617	2617	2617	2617	2617	2617

Z values are in parentheses. ***, **, and * indicate significance at the levels of 1%, 5%, and 10%, respectively.

**Table 12:** Marginal effects of the main variables under different thresholds.

Variable	Pr (y = 1)	Pr (y = 2)	Pr (y = 3)
Model 1			
RPDH × lnoperate	−0.001	−0.007 ***	0.008 ***
	(0.001)	(0.002)	(0.002)
Control	Yes	Yes	Yes
Model 2			
RPDH × lnsalary	−0.001	−0.005 ***	0.006 ***
	(0.001)	(0.002)	(0.002)
Control	Yes	Yes	Yes
Model 3			
RPDH × lnperincome	−0.001	−0.007 ***	0.008 ***
	(0.001)	(0.002)	(0.002)
Control	Yes	Yes	Yes

Delt-method standard errors are in parentheses. *** indicates significance at the levels of 1%.

## Data Availability

The datasets used and/or analyzed during the current study are available to the public on reasonable request. Please contact the corresponding author.

## Abbreviations

RPDH	Renovation Program of Dilapidated Houses
PSM	Propensity Score Matching
ATT	Average Treatment effect on the Treated
